# Interactions between boldness, foraging performance and behavioural plasticity across social contexts

**DOI:** 10.1007/s00265-016-2193-0

**Published:** 2016-08-04

**Authors:** Guðbjörg Ásta Ólafsdóttir, Kit Magellan

**Affiliations:** 1 Research Centre of the Westfjords, University of Iceland, Adalstraeti 21, IS415 Bolungarvík, Iceland; 2 School of Biological Sciences, University of Hong Kong, Pokfulam, Hong Kong; 3 South African Institute for Aquatic Biodiversity, Private Bag 1015, Grahamstown, 6139 South Africa

**Keywords:** Cognitive style, Threespine stickleback, Behavioural plasticity, Boldness, Audience effect, Speed-accuracy trade off

## Abstract

**Abstract:**

Boldness, the tendency to be explorative, risk prone and proactive, often varies consistently between individuals. An individual’s position on the boldness–shyness continuum has many implications. Bold individuals may outperform shyer conspecifics during foraging as they cover more ground, accumulate information more rapidly and make more frequent food discoveries. Individual variation in boldness may also affect behavioural plasticity across environmental contexts, as the time to process new information, the ability to locate and memorise resources and the time and ability to apply prior information in a novel context all differ between individuals. The primary aim of the current study was to examine plasticity in, and covariation between, boldness, foraging speed and foraging accuracy across social foraging contexts. We showed that the stickleback that were shyest when foraging alone became relatively boldest when foraging in a social context and also delayed their entry to a known food patch the most in the presence of conspecifics. These results support the assertion that shyer foragers are more reactive to social cues and add to current knowledge of how an individual’s position on the boldness–shyness continuum may correlate to foraging task performance and behavioural plasticity. We conclude that the correlation between boldness and behavioural plasticity may have broad relevance as the ability to adjust or retain behaviours in changing social environments could often have consequences for fitness.

**Significance statement:**

Animal personality may affect how much individuals change their behaviour to suit different environments. We studied the link between threespine stickleback personality (boldness or shyness), foraging performance and change in foraging performance when either alone or in the presence of other stickleback. We found that shyer threespine stickleback were more reactive to the presence of other fish when foraging. When observed or joined by other fish, shy stickleback started exploring earlier, but entered a known food patch later, than when alone. Bolder stickleback changed their foraging behaviour much less in the presence of other fish. Our results suggest that how bold or shy individuals are may have important consequences on how well they adjust their foraging behaviour to environmental change.

**Electronic supplementary material:**

The online version of this article (doi:10.1007/s00265-016-2193-0) contains supplementary material, which is available to authorized users.

## Introduction

Consistent variation in individual behaviour across contexts and behavioural trait correlation are often termed animal personality and behavioural syndromes (Dingemanse et al. 2003; Dall et al. 2004; Sih et al. 2004; Réale et al. 2007). Boldness, the tendency to be explorative, risk prone and proactive, is a much studied behavioural trait that often varies consistently between individuals and is correlated with other behavioural traits, such as aggression and activity (Huntingford 1976; Coleman and Wilson 1998; Biro and Stamps 2008). An individual’s position on the boldness–shyness continuum (Wilson et al. 1994) has many implications: bold individuals may, for example, outperform shyer conspecifics during foraging as they cover more ground, thereby accumulating information more rapidly and making more frequent food discoveries. However, speed may also come at the cost of accuracy as individuals that make rapid decisions may not weigh all relevant data (Chittka et al. 2003; Sih and Del Giudice 2012; Moiron et al. 2016).

Traits such as the speed and accuracy of locating food resources have been quantified and discussed in relation to cognitive abilities (Burns and Rodd 2008; Ducatez et al. 2015; Mamuneas et al. 2015). As cognition relates to how information is acquired, processed, stored or acted upon, it is likely to be central to many evolutionary and behavioural ecology processes (Sih and Del Giudice 2012). However, cognitive processes in animals may be difficult to quantify (Griffin et al. 2015). Individual variation in task performance may be a useful indicator of cognitive abilities without any assumptions as to the proximate cause. Task performance has been correlated with boldness in previous studies. For example, the performance accuracy of black-capped chickadees covaried with exploratory behaviour (Guillette et al. 2015). Conversely, a recent study on threespine stickleback found that bold individuals were no less accurate than shy ones in a t-maze foraging task (Mamuneas et al. 2015).

Concomitant with individually consistent traits is individual behavioural plasticity. The correlation between boldness, task performance and behavioural plasticity has received limited empirical attention although between-individual variation in both cognitive processing and boldness may be expected to affect behavioural plasticity across environmental contexts. For example, the time to process new information, the ability to locate and memorise resources and the time and ability to apply prior information in a novel context all differ between individuals. Examining variation on the boldness–shyness continuum within the framework of coping styles (Coppens et al. 2010) allows useful deductions about the correlation between behavioural plasticity, boldness and task performance (Koolhaas et al. 2007; Coppens et al. 2010; Sih and Del Giudice, 2012). To the extent that boldness corresponds to proactivity (Koolhaas et al. 2007), bold individuals would be expected to be less sensitive to changes in the environment and more likely to form inflexible routines (Benus et al. 1990; Coppens et al. 2010). This in turn would cause bolder individuals to be less behaviourally plastic, in particular when an added level of complexity, or stimuli, is added to a previously familiar environment or a mastered task. This may often be relevant to foraging contexts, for example, when foraging responses are adjusted to the presence of others, as the regulation and timing of responses is likely to depend on both individual sensitivity to the environment and cognitive processing, such as in cases of audience effects (Bugnyar and Heinrich 2006; Shaw and Clayton 2013).

The primary aim of the current study was to examine (1) plasticity in, and (2) covariation between, boldness (latency to explore), foraging speed (latency to feed from a known food patch) and foraging accuracy (choosing a full food patch over an empty patch), across social foraging contexts. Specifically, we measured latency to explore, latency to feed and accuracy of food patch choice in individual threespine stickleback in a solitary foraging task over seven consecutive days. Food was placed in the same position throughout, allowing individuals to accumulate knowledge on food location. We then examined behavioural plasticity in the same traits across social contexts, by having focal individuals repeat the same foraging task while being either observed or joined by conspecifics. We predicted that bolder individuals would be faster, but less accurate, than their shyer conspecifics during foraging. We also predicted that bold foragers would be more proactive in the presence of conspecifics, that is, either increase their foraging speed and accuracy, or retain prior performance, while shyer individuals would become relatively slower at completing the foraging task as they would be more sensitive to the change in social context, thereby taking longer to process the added social information and subsequently apply their prior foraging knowledge.

## Methods

### Study subjects and housing

We used a wild population of threespine sticklebacks, *Gasterosteus aculeatus*, caught with unbaited minnow traps from a vegetated freshwater lake in October 2014 (Syðridalsvatn, 66° 7’ N, 23° 14’ W). Fish were housed in an aerated and filtered gravel-lined holding aquarium (150 × 40 × 30 cm). Temperature was kept at 12.5 °C, with an 8:16 h light/dark cycle regime. Fish were fed defrosted frozen bloodworms (*Chironomus* spp.) to satiation daily. Experiments took place in November and December 2014. Twenty-four focal fish were randomly chosen and placed in individual, gravel-lined 48-L tanks, with a single rock (c.a. 100 cm^3^) placed in one corner for refuge and to facilitate orientation. All tanks were continuous flow through, filtered and aerated. After a single day of acclimating to experimental conditions in the individual home tanks, we began observations. We were able to unambiguously identify each focal fish as they were subjected to all of the experimental tasks below. To minimise observer bias, data were analysed blind by assigning all recorded videos randomly scrambled numbers (including information on relative size of focal fish—joiner fish in social task 2) before extracting data. It was not possible to prevent the single observer from recognising individual focal fish by other means.

### Experimental protocols

#### Experiment one: solitary foraging task

**Fig. 1:**
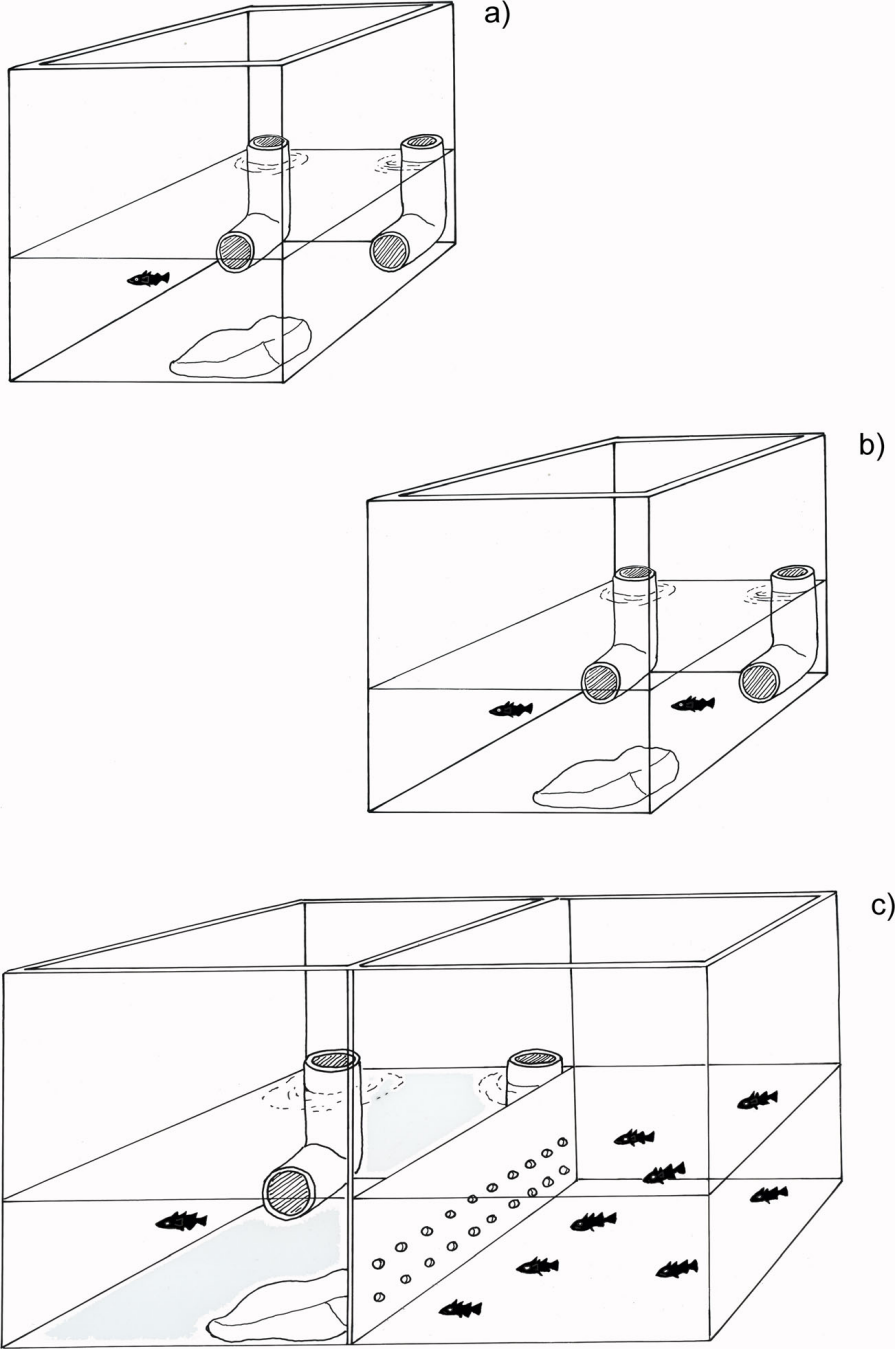
Experimental set-up. **a** Solitary foraging task. **b** Social foraging task 2: joined by a conspecific. **c** Social foraging task 1: observed by conspecifics

The solitary foraging task aimed to assess individual variation in latency to explore, latency to discover and locate a spatially consistent food source and accuracy in locating a spatially consistent food source. Within their home tank (Fig. 1a), the stickleback were first gently herded to the rock that thus served as a starting point. Two opaque plastic tubes were then placed in opposite corners of each tank. The tubes had a right angle at the bottom so that food did not fall out and fish had to enter the tube to feed (Ólafsdóttir et al. 2014). One tube, representing the food patch, contained c.a. 5 g of defrosted bloodworms. The inside of the other tube was smeared with liquid from the defrosted bloodworms to reduce the effects of olfactory cues in tube choice. Throughout the experiments, focal fish were fed to satiation each day using the tubes and received no other food. On day 1, the tubes were left for 2 h, during which time most of the fish discovered the food location (20 of 24). Day one was intended to make the focal fish aware of the position of the food and no behavioural measures were made. The four fish that did not discover the food on day one all entered the feeder on day two. Two were used in subsequent analysis but two were excluded from the statistical analyses (see explanation below). The tubes were placed as before for seven consecutive days (Trials 1–7) with food being presented in the same position throughout. From 20-min videos recorded at the beginning of each trial on the 7 days, we extracted (1) “boldness” defined as latency to explore, i.e. move away from the rock, after tubes were placed; (2) “foraging speed” defined as time to food detection and location, i.e. latency to enter the feeder tube and feed after commencing exploration (we did not use total time before entering the feeder to avoid a direct effect of boldness on this measure) and (3) “foraging accuracy” defined as correct entry, i.e. entering the feeding tube before the control tube. The fish needed to enter the tube by c.a. half a body length to gain the food reward and this distance defined an entry event for both tubes. The rock had been purposefully placed in the tank corner furthest from the feeding tube to reduce the likelihood of correct entry by chance, i.e. the fish had to move further from the rock to enter the feeder tube than the control tube (Fig. 1).

#### Experiment two: behavioural plasticity across social foraging contexts

Following the solitary foraging task, each focal fish underwent two consecutive foraging tasks in a setting identical to the solitary foraging task but with different social contexts. Two measures were obtained for each individual in Task 1 and three in Task 2, in random order, over a period of 3 days. These tasks were aimed at measuring individual behavioural plasticity in boldness and foraging performance across social contexts but in the specific context of prior knowledge of food location.

For Task 1, each focal fish was transferred to an empty compartment, identical to the individual test tanks but with a transparent tank divider to a neighbouring compartment, which allowed both visual and olfactory cues (Fig. 1c). We did not use the individual’s home tank to minimise the effects of residency or dominance that had been observed previously in a similar setting (Ólafsdóttir et al. 2014). The adjacent compartment contained a small shoal of stickleback (10 individuals) previously unknown to the focal fish. The focal fish was allowed to acclimate to the new tank for 10 min after which the tubes where placed as before. For task 2, the focal fish, together with a naive partner, was transferred to an empty compartment (Fig. 1b) and allowed to acclimate for 10 min after which food was supplied using the same feeding protocol as in task 1. During task 2, the focal fish and its partner could be identified by small size differences (<3 mm), each focal fish was tested with both smaller and larger partners and partner size did not affect focal fish behaviour (see supplementary material). The naïve partner never entered the feeding tube before the focal fish.

During tasks 1 and 2, the feeding tube contained only a single bloodworm to minimise effects on feeding motivation while retaining a reward for correct entry. The fish were then fed in their home tanks at the end of these tasks. The control tube remained empty. Each trial was videoed for 10 min (as experience from experiment 1 suggested that most fish completed the task within this time) and (1) boldness, (2) foraging speed and (3) foraging accuracy assessed from videos as before.

### Statistical analysis

Two individuals were inactive for most of the tasks; their data was therefore not used for statistical analyses, resulting in a final sample size of *n* = 22. Data from trial 4 of the solitary foraging task was missing for six individuals due to equipment failure; data from this trial was therefore excluded from all analyses, resulting in six behavioural measures for each individual in a solitary context (total number of solitary observations, *n* = 132) and five in the two social contexts (total number of social task observations, task 1 *n* = 44; task 2 *n* = 66). We used the software R v. 3.1.3 (http://www.r-project.org) (R Core Team 2012) for all statistical analyses.

#### General model specifications

We used a Bayesian framework using Markov chain Monte Carlo (MCMC) methods in the R package MCMCglmm (Hadfield 2010) for both random regression (RR) models, examining behavioural plasticity, and bi-variate general linear mixed models (GLMM), to examine trait covariation. Residual variance (or within-individual variance, V_e_) could not be estimated for the binomial trait of correct food patch choice (and was fixed at 1.0 in all models). We therefore did not attempt to simultaneously estimate effects for all traits and trait correlation in a multivariate model but constructed univariate RR models to examine behavioural change within and across contexts, and bi-variate GLMMs to examine trait correlation. In all analyses, the dependent variables of boldness (latency to explore) and foraging speed (latency to feed) were modelled using a Poisson distribution with log link function and accounting for overdispersion with an additive model (Hadfield 2015), and foraging accuracy (correct food patch choice) was modelled with a binomial distribution with a probit link function. In all models, we used non-informative priors (inverse Wishart) for both individual and residual variance of latency to explore and latency to feed and individual variance of correct food patch choice (using informative priors had little or no effect on model estimates). All models were run with a burn-in of 1.000.000 and subsequent 5.000.000 iterations and a thinning interval of 1.000. We visually inspected plots of the traces and posterior distributions and calculated the autocorrelation between samples to make sure that the models converged. Autocorrelation was <0.02 and effective sample size was ∼4.000 for all estimates. We inspected the 95 % highest posterior density (HPD) associated with each fixed effect, between-individual variance, within-individual variance, covariation and the repeatability estimates to check whether they overlapped with zero. A 95 % HPD interval contains most of the posterior distribution and is analogous to a confidence interval in the frequentist approach; a 95 % HPD that overlaps 0 indicates that the effect does not differ significantly from zero (Hadfield 2010; Dingemanse and Dochtermann 2013).

#### Random regression models

We performed two sets of random regression (RR) analyses. First, we examined whether the three behaviours changed across trials within the solitary foraging task (experiment 1), whether individuals differed in their level of change of each behaviour and whether initial behaviour and behavioural change across trials correlated. For this analysis, we included trial number as a (numerical) fixed effect, individual identity as a random effect, allowed individual slopes to vary across trials and also estimated the intercept–slope covariation. The x variable (trial number) was centred on the first day of the solitary task, meaning that the intercept, and intercept–slope covariation, should be taken to indicate behaviour, or covariation of behaviour, at the onset of the experiment. In the case of significant covariation, we calculated intercept–slope correlation by dividing the covariance by the square root of the product of the variance.

**Fig. 2:**
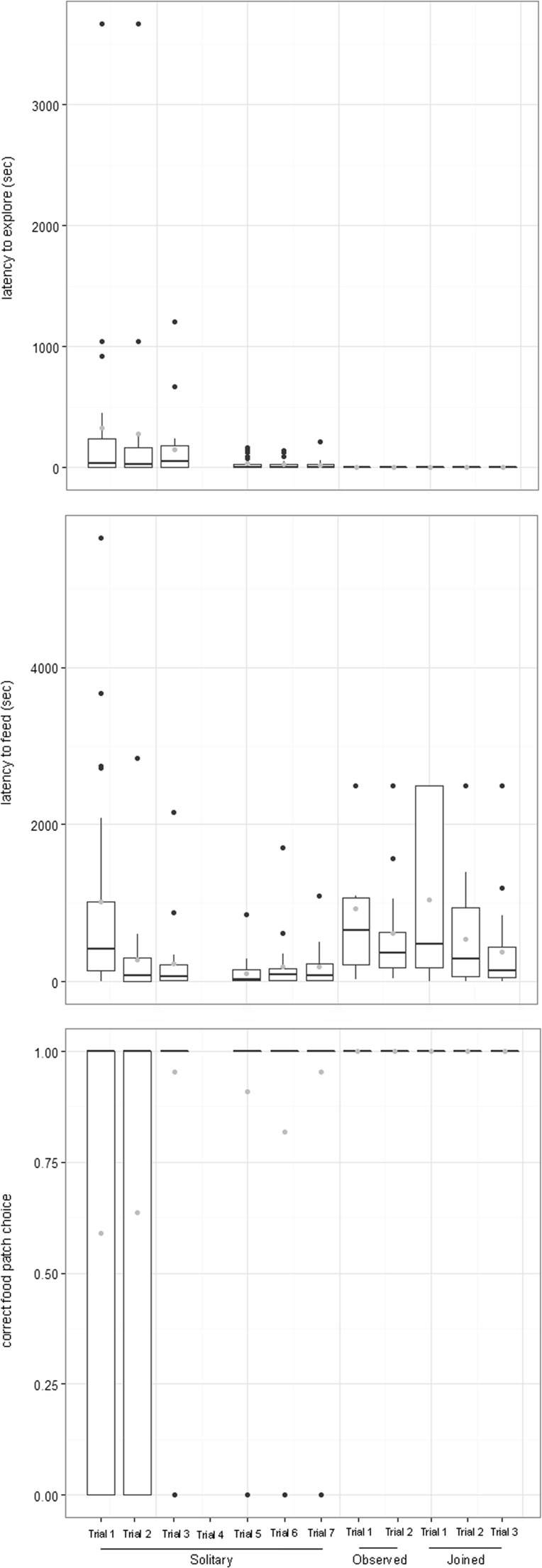
Depiction of raw data showing boldness (latency to explore in seconds), foraging speed (latency to feed in seconds) and correct food patch choice across trials of the solitary foraging task and social foraging tasks (task 1: observed by conspecifics and task 2: joined by conspecifics). *Boxplots* depict median (*horizontal black line*), first (*box*) and third (*whiskers*) quartile. Values falling out with the third quartile are presented as *black circles* and *grey circles* represent mean values

Second, we examined whether behaviours changed across foraging tasks (experiments 1 and 2), whether individuals differed in their level of change across tasks and whether mean level behaviour during the solitary foraging task and behavioural change correlated across tasks. Different approaches could be taken to examine behavioural plasticity across the foraging tasks. We expected (and observed) changes in all behavioural traits across trials of the solitary foraging task, as individuals became familiar with the foraging setting. Examining behavioural slopes between only the last few trials of the solitary foraging task and the two social foraging tasks would represent how much individual behaviour was altered by the added complexity of social context after becoming familiar with the foraging setting. However, we chose to analyse the complete data set (including all measures from the solitary foraging task) as information on between-individual variance would be lost by excluding the first trials of the solitary foraging task (between-individual variance in behaviour decreased in later trials of the solitary foraging task; Fig. 2). We constructed three univariate models. Each model included trial number nested within social foraging task as fixed effects and individual identity as a random effect allowing individual slopes to vary across social foraging tasks and estimating intercept–slope covariation (reflecting covariation between the solitary task mean and slope across tasks). We calculated intercept–slope correlation from significant covariation by dividing the covariance by the square root of the product of the variance. A priori, we expected negative intercept–slope correlations across tasks (as any individual that by chance draws a high value in the first task draws a relatively lower value in the second task and vice versa). Therefore, we also calculated the correlation of individual behaviour in the solitary foraging task and each of the social foraging tasks by first extracting the individual intercepts in the social foraging task and calculating the individual intercepts in each of task 1 and task 2 as individual intercept + 2 × individual slope and then calculating the correlation between these values.

To include a standardised metric of behavioural consistency, and therefore personality, and give greater confidence to other interpretations, we calculated repeatability for each trait (latency to explore, latency to feed and correct food patch choice) as the ratio of between-individual variance (V_ind_) over the sum of V_ind_ and the residual variance (V_e_) from the random regression models (both within the solitary foraging context and across contexts) (Nussey et al. 2007; Dingemanse et al. 2010; Dingemanse and Dochtermann 2013). The results are reported in the electronic supplementary material.

#### Behavioural trait covariation and correlation

To determine whether latency to explore, latency to feed and correct food patch choice were correlated with one another, we used bi-variate mixed effect models within MCMCglmm (Hadfield 2010), following the guidelines by Dingemanse and Dochtermann (2013). We conducted separate models for covariation within the solitary foraging task and between solitary and social foraging tasks. In models using data from the solitary foraging task, the fixed effect was trial number and individual identity a random effect. In models using data across social foraging trials, the fixed effects in each model were trial number nested within social context and individual identity was a random effect. In all bi-variate models, we allowed heterogeneous variance of behavioural traits and estimated covariance matrices (using the us function in MCMCglmm; Hadfield 2015). We calculated between- and within-individual correlation from significant covariance by dividing the covariance of the traits in question by the square root of the product of their variance.

## Results

During the solitary foraging task, most individuals rapidly discovered the food patch after which they consistently entered the correct food patch (Fig. 2). Mean foraging accuracy (correct food patch choice) across all solitary task trials was 0.81 (SD = 0.39). Mean boldness (latency to explore) was 141.18 (SD = 479.67) across all solitary task trials, 1.15 (SD = 0.60) in task 1 (observed) and 1.19 (SD = 0.48) in task 2 (joined). Mean foraging speed (latency to feed) was 473.75 (SD = 883.46) across all solitary task trials, 653.52 (SD = 830.30) in task 1 (observed) and 771.34 (SD = 830.43) in task 2 (joined). All times are in seconds.

**Fig. 3:**
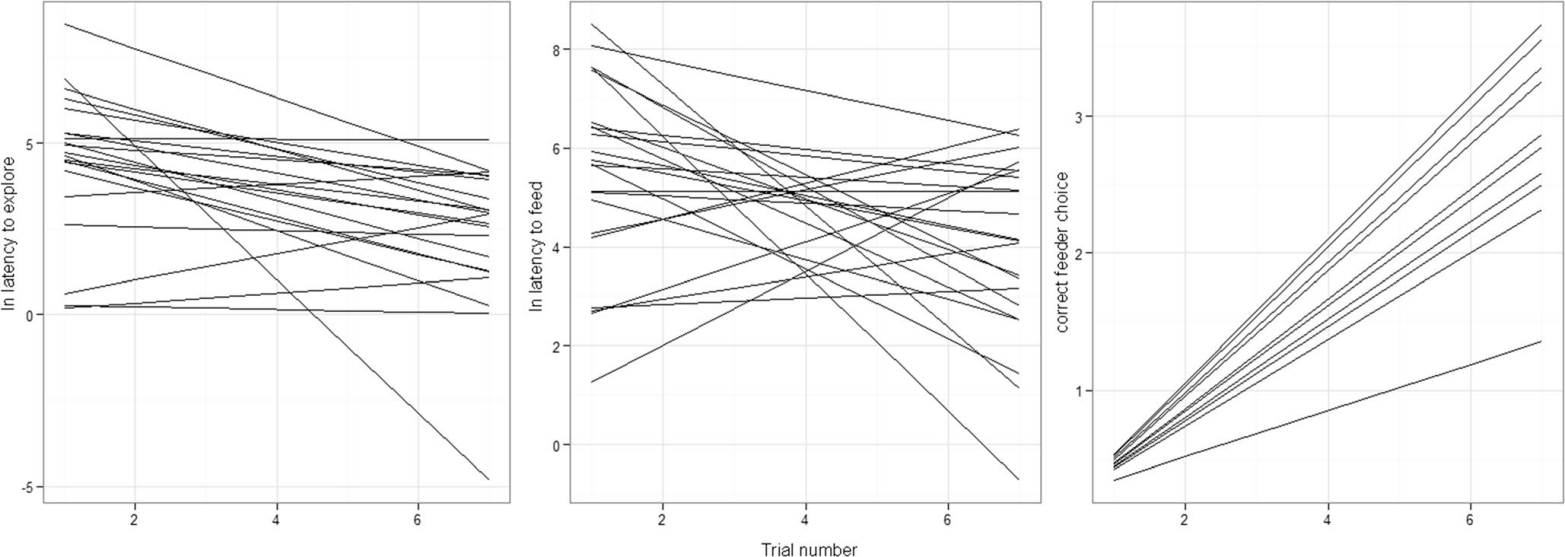
*Lines* represent fitted individual slopes of boldness, foraging speed and correct entry across trials of the solitary foraging task

Results from the RR analysis within the solitary foraging task showed that trial number significantly affected boldness and foraging accuracy, individuals became bolder and more accurate across trials (Figs. 2 and 3). The RR analysis also showed significant between-individual variation in boldness, while for foraging accuracy both the intercept and the slope showed significant between-individual variation. Neither individual intercept nor slope differed for foraging speed nor did individuals increase or decrease foraging speed across trials. For no trait was there significant individual intercept and slope covariation, that is, the initially boldest, fastest and most accurate foragers did not improve/change most (or least) during solitary foraging (Table 1). Because of the relatively low sample size (number of individuals vs. observation number), the current study may lack power to detect smaller effects on individual intercept–slope covariation (Martin et al. 2011; van de Pol 2012); non-significance should therefore be interpreted with care.

**Table 1:** Results of the random regression (RR) models examining between- and within-individual variation in boldness (latency to explore), foraging speed (latency to feed) and foraging accuracy (correct food patch choice), between-individual variation in the slopes of those traits across trials as well as mean–slope covariation across trials

		Posterior mode	95 % HPD interval	
Latency to explore	Random structure			
	ID intercept	**4.444**	0.502	9.490
	Intercept/slope covariation	−0.276	−0.895	0.167
	ID slope	0.031	0.000	0.098
	Within-individual variation	**3.560**	2.529	4.670
	Fixed effects			
	Model intercept	3.560	2.529	4.670
	Trial number	−**0.329**	−0.499	−0.155
Latency to feed	Random structure			
	ID intercept	2.616	0.000	6.845
	Intercept/slope covariation	−0.386	−1115	0.057
	ID slope	0.068	0.000	0.198
	Within-individual variation	**3.818**	2.687	5.037
	Fixed effects			
	Model intercept	**4.777**	3.798	5.739
	Trial number	−0.168	−0.360	0.021
Correct food patch choice	Random structure			
	ID intercept	**1.384**	0.322	3.139
	Intercept/slope covariation	−0.262	−1.008	0.323
	ID slope	**0.706**	0.252	1.305
	Within-individual variation	–	–	–
	Fixed effects			
	Model intercept	−0.444	−1.826	0.834
	Trial number	**0.991**	0.431	1.594

Boldface indicates estimates were the 95 % HPD interval does not overlap with 0

**Fig. 4:**
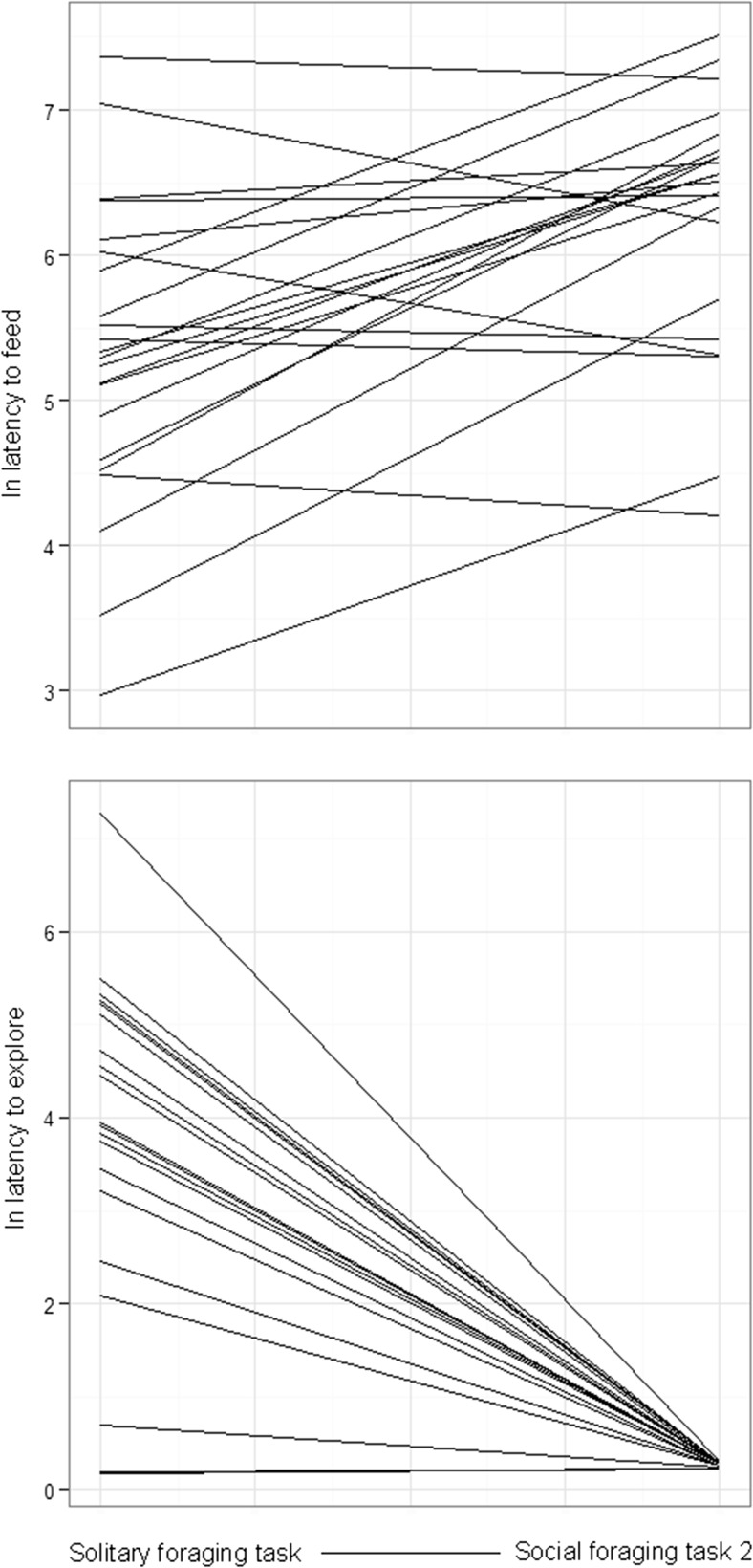
*Lines* represent fitted individual slopes of boldness and foraging speed across the solitary foraging task and social foraging task 2 (joined by conspecifics). Solitary foraging task 1 was not shown as the effect was similar but less pronounced than for task 2

Results from the RR analysis across social foraging tasks showed that boldness increased across social foraging tasks (from solitary to either observed or joined) and foraging speed decreased (Fig. 4), but this was only significant between the solitary foraging task and social task 2 (joined by a conspecific) (Table 2). There was significant between-individual variation in both boldness and foraging speed across social contexts and significant variation in individual slopes across social contexts. Individual intercept and slope of boldness were negatively correlated (Table 2) and there were negative correlations between individual boldness in the social foraging task and boldness in each of task 1 and task 2 (*r* = −0.73, *p* < 0.01 and *r* = −0.75, *p* < 0.01, respectively). These results support that shyer individuals became relatively bolder in a social context (primarily reflecting between individual variation in boldness in the solitary task as all individuals were bold in the social tasks, Fig. 2) and that the correlation is not a statistical artefact caused by regression to the mean (as discussed in the methods section). Conversely, individual mean values of foraging speed in the solitary foraging task were positively correlated with foraging speed in each of task 1 and task 2 (*r* = 0.58, *p* < 0.01 and *r* = 0.72, *p* < 0.01, respectively), suggesting that the slight (non-significant) negative covariation of foraging speed intercept and slope (Table 2) was a statistical artefact.

**Table 2:** Results of the random regression (RR) models, across social foraging tasks, examining between- and within-individual variation in boldness (latency to explore) and foraging speed (latency to feed), between-individual variation in the slopes of those traits across tasks as well as intercept–slope covariation across tasks

			Posterior mode	95 % HPD interval	
Latency to explore	Random structure	ID intercept	**2.326**	0.987	0.040
		Intercept/slope covariation, task 2	**−1.828**	**−**3.448	**−**0.413
		Intercept/slope correlation, task 2	**−0.819**	**−**0.931	**−**0.451
		ID slope, task 2	**2.314**	0.681	4.355
		Intercept/slope covariation, task 1	**−1.772**	**−**3.529	**−**0.413
		Intercept/slope correlation, Task 1	**−0.823**	**−**0.940	**−**0.562
		ID slope, task 1	**2.317**	0.583	4.401
		Within-individual variation	**2.116**	1.637	2.611
	Fixed effects	Model intercept	**3.771**	2.947	4.605
		Task 2	**−4.001**	**−**5.436	**−**2.616
		Task 1	**−3.696**	**−**5.485	**−**1.764
		Solitary task: trial number	**−0.338**	**−**0.467	**−**0.217
		Task 2: trial number	0.062	**−**0.435	0.595
		Task 1: trial number	**−**0.120	**−**1.214	0.918
Latency to feed	Random structure	ID intercept	**0.922**	0.369	1.632
		Intercept/slope covariation, task 2	**−**0.169	**−**0.811	0.407
		ID slope, task 2	**1.068**	0.344	2.079
		Intercept/slope covariation, task 1	**−**0.124	**−**0.701	0.368
		ID slope, task 1	**0.819**	0.275	1.527
		Within-individual variation	**2.980**	2.366	3.601
	Fixed effects	Model intercept	**4.820**	4.107	5.596
		Task 2	**1.327**	0.001	2.666
		Task 1	1.670	**−**0.095	3.487
		Solitary task: trial number	**−0.173**	**−**0.317	**−**0.045
		Task 2: trial number	**−**0.398	**−**0.909	0.110
		Task 1: trial number	**−**0.335	**−**1354	0.705

Only significant intercept–slope correlations are shown. Boldface indicates estimates were the 95 % HPD interval did not overlap with 0

There were significant between-individual correlations for foraging speed and foraging accuracy, both within the solitary foraging task and across the social foraging tasks. Fast foragers were also more accurate (Table 3). Note however that when joined or observed by conspecifics all individuals chose the correct food patch. There were significant between- and within-individual correlations for boldness and foraging speed, but only across the social foraging tasks. Individual boldness correlated negatively with foraging speed: the individuals that started exploring fastest across social contexts also delayed their entry to the food patch the most (Table 3).

**Table 3:** Covariation and correlation of boldness (latency to explore), foraging speed (latency to feed) and foraging accuracy (correct food patch choice) across social tasks and across trials within the solitary foraging task

		Across social tasks			Within solitary foraging		
		Posterior mode	95 % HPD interval		Posterior mode	95 % HPD interval	
Latency to explore/latency to feed							
Between individuals	Covariation	−2.32	−5.49	−1.03			
	Correlation	−0.85	−0.96	−0.56			
Within individuals	Covariation	−0.70	−1.46	−0.22			
	Correlation	−0.20	−0.33	−0.06			
Latency to feed/correct food patch choice							
Between individuals	Covariation	−1.18	−3.30	−0.26	−1.63	−4.02	−0.25
	Correlation	−0.79	−0.95	−0.23	−0.81	−0.96	−0.27
Within individuals	–	–	–	–	–	–	–

Only effects where the 95 % HPD interval did not overlap 0 are shown. Full model results can be found in the electronic supplementary material

## Discussion

We have shown that the degree of individual change in both boldness and foraging speed differs across social foraging contexts and that the level of an individual’s boldness correlated negatively with the level of change in the same trait - individuals that were shyer in the solitary foraging task became relatively bolder in the social foraging tasks. Boldness and foraging speed were negatively correlated across social foraging contexts - the individuals that became relatively boldest in the social task also delayed their entry to a known food patch the most in the presence of conspecifics. Both of these results support the assertion that shy foragers are more reactive to social cues and add substantially to the current knowledge of how an individual’s position on the boldness–shyness continuum may correlate to foraging task performance and behavioural plasticity, particularly across contexts that vary in sensory or social complexity.

Foraging performance includes both foraging speed and accuracy. Boldness and foraging performance may be expected to correlate for a number of reasons. Cognitive processing requires gathering information, recalling past memory and processing cues from the immediate environment, all of which are likely to depend in part on how much ground the individual covers in a given time. However, contrasting predictions ensue as speed may also result in more frequent errors or less careful information processing (Chittka et al. 2003; Mathot et al. 2012; Sih and Del Giudice 2012; Moiron et al. 2016). In the current study, boldness correlated with neither foraging speed nor foraging accuracy within the solitary foraging task, suggesting that boldness was not beneficial to task performance in this setting. These results are in concordance with a recent study, also on threespine stickleback, where boldness—measured as time of exploration and catchability—and correct choice in a t- maze foraging task did not correlate (Mamuneas et al. 2015). Correct food patch choice and foraging speed were however correlated in the current study; faster foragers were also more likely to choose the correct food patch (Table 3), suggesting that both traits adequately reflect task proficiency.

Several previous studies have found correlation between animal personality and behavioural plasticity across contexts (individual intercept–slope covariation) (Mathot et al. 2012; Yuen et al. 2015; Fürtbauer et al. 2015). Aggressive mice, for example, do not change their level of aggression across social context, whereas less aggressive mice do (Natarajan et al. 2009). Most individuals in the current study started exploring faster in the social contexts (Fig. 4). Behavioural plasticity in boldness across social foraging contexts correlated with boldness during the solitary foraging task (Table 2) and the negative correlation between individual boldness during solitary foraging and boldness in the social foraging tasks supports that this is not a statistical artefact. We therefore conclude that shyer foragers started exploring relatively quicker when observed or joined by conspecifics whereas bolder individuals retained their latency to explore (Fig. 4, Table 2). In fact, most individuals rapidly started exploring in the presence of conspecifics (Fig. 4). Conversely, most individuals were slower to enter the feeder when observed or joined by conspecifics than when foraging alone and foraging speed in the solitary foraging task was correlated to foraging speed in the social tasks. However, change in boldness and change in foraging speed correlated across social foraging contexts (Table 3), suggesting that the shyer individuals—that became relatively boldest across contexts—also delayed their entry to the feeder most in conspecific presence. It has been suggested that the most extreme individuals, in either boldness or shyness, would be less sensitive to change (Sih and Del Giudice 2012) but also that bold individuals may display a more proactive reaction to stress, stimuli or change (Koolhaas et al. 2007). The current results concur with the latter prediction.

Showing consideration of social context during foraging may also represent reluctance to share resource information, i.e. an audience effect. Audience effects occur in different contexts, including during aggressive interactions and courtship (Doutrelant et al. 2001; Auld and Godin 2015). In a foraging context, pilfering ravens and jays may, for example, attempt to reduce foraging information available to conspecifics to protect their catch (Emery and Clayton 2001; Bugnyar and Heinrich 2006; Shaw and Clayton 2013). The greater delay in entering the food patch by shyer individuals observed in the current study may signal more active reluctance to share information but could also indicate that shy individuals are more reactive to change and take longer to recognise and process foraging information in a changed social context. The consequence of either effect would be that bolder, faster learning individuals would be more likely to produce foraging information for conspecifics, which is in line with a recent study on house sparrows (Katsnelson et al. 2011).

We conclude that while there were no differences in the solitary foraging performance of shy and bold individuals, shyer individuals were more reactive to social cues during foraging. Behavioural plasticity may determine persistence and selection in changing environments (Sih et al. 2011) and examining how position on the boldness–shyness continuum may affect task performance, and behavioural plasticity in task performance, across changing environments is of current interest. Although the current experiment presents a very specific case of behavioural plasticity, in how foraging knowledge is applied across social context, it may well be argued that the scenario has broad relevance. In nature, individuals become familiar with a foraging landscape and the ability to adjust or retain behaviours (behavioural plasticity) to changing social environments within that landscape could often have consequences for fitness.

## Electronic supplementary material

ESM 1 (DOCX 46 kb)
