# Supplementary material for: Interactions between boldness, foraging performance and behavioural plasticity across social contexts
Source: Behav Ecol Sociobiol. 2016 Aug 4;70(11):1879–89. doi: 10.1007/s00265-016-2193-0 (PMC5054052; doi:10.1007/s00265-016-2193-0)
Supplement: Supplementary file 1 — (DOCX 46 kb) [file 265_2016_2193_MOESM1_ESM.docx]

**Electronic Supplementary Material**

Interactions between boldness, foraging performance and behavioural plasticity across social contexts

Behavioral Ecology and Sociobiology

Guðbjörg Ásta Ólafsdóttir^*^ & Kit Magellan

*corresponding author: gaol@hi.is

**Supplement to experiments**

Small size differences were used to differentiate the focal fish from the naïve joiner during social foraging task 2. Each focal fish was tested once with a slightly larger conspecific and twice with a slightly smaller conspecific (size difference 1.0-3.0 mm). Ideally all fish would have been tested with same size naïve partners or alternatively naïve partners with equal size differences but we did not have access to enough naïve fish to make this possible. We used a generalized linear model to examine the potential effect of size difference on latency to explore and latency to feed. We ran two models using each of latency to explore or latency to feed as the dependant variable, size difference as a fixed effect and both ID and trial number as random effects. Both traits were modelled with a Poisson distribution. Size difference did not significantly affect either trait (latency to explore, p = 0.41; latency to feed p = 0.179).

**Supplementary table 1**. Repeatability estimates calculated from random regression models (presented in tables 1 & 2 of the full text).

|  | posterior mode | 95% HPD interval | |
| --- | --- | --- | --- |
| Within the solitary foraging context |  |  |  |
| latency to explore | 0,56 | 0,17 | 0,67 |
| latency to feed | 0,41 | 0,00 | 0,58 |
| correct feeder choice | 0,58 | 0,24 | 0,76 |
| Across social foraging contexts |  |  |  |
| latency to explore | 0,52 | 0,38 | 0,02 |
| latency to feed | 0,24 | 0,13 | 0,31 |

**Supplementary table 2.** Full results of the bi-variate GLMM. Significant covariations are summarized in table 2 of the full text.

|  | Across social foraging tasks | | |  | Within the solitary foraging task | | |
| --- | --- | --- | --- | --- | --- | --- | --- |
|  | posterior mode | 95% HPD interval | |  | posterior mode | 95% HPD interval | |
| Latency to explore ID | 0,85 | 0,50 | 2,07 | Latency to explore ID | 2,79 | 1,45 | 5,75 |
| Covariation ID | -0,29 | -0,98 | 0,08 | Covariation ID | -0,79 | -2,17 | 0,07 |
| Correct feeder choice ID | 0,39 | 0,10 | 1,11 | Correct feeder choice ID | 0,63 | 0,14 | 1,85 |
| Intercept | 3,41 | 2,74 | 4,05 | Intercept | 2,60 | 2,13 | 3,14 |
| Task 2 | 1,31 | 1,02 | 1,60 | Trial number | -0,21 | -0,28 | -0,13 |
| Task 1 | 1,86 | 1,52 | 2,21 |  |  |  |  |
| Latency to feed ID | 0,88 | 0,41 | 3,21 | Latency to feed ID | 1,24 | 0,48 | 4,22 |
| Covariation ID | -1,18 | -3,30 | -0,26 | Covariation ID | -1,63 | -4,02 | -0,25 |
| Correct feeder choice ID | 3,27 | 0,81 | 9,01 | Correct feeder choice ID | 3,02 | 0,83 | 10,23 |
| Intercept | 2,62 | 2,31 | 2,91 | Intercept | 3,72 | 2,89 | 4,53 |
| Task 2 | -1,84 | -2,19 | -1,54 | Trial number | -0,08 | -0,16 | -0,01 |
| Task 1 | -1,84 | -2,24 | -1,46 |  |  |  |  |
| Latency to explore ID | 3,52 | 1,03 | 9,80 | Latency to explore ID | 4,02 | 1,31 | 7,48 |
| Covariation ID | -2,32 | -5,49 | -1,03 | Covariation ID | -1,03 | -2,55 | 0,21 |
| Latency to feed | 2,81 | 0,50 | 7,32 | Latency to feed | 1,04 | 0,19 | 2,24 |
| Latency to explore units | 3,83 | 3,20 | 5,20 | Latency to explore units | 3,64 | 2,58 | 4,75 |
| Covariation units | -0,70 | -1,46 | -0,22 | Covariation units | 0,47 | -0,29 | 1,29 |
| Latency to feed units | 4,13 | 3,32 | 5,27 | Latency to feed units | 4,09 | 2,94 | 5,24 |
| Intercept | 3,37 | 2,50 | 4,16 | Intercept | 4,55 | 3,90 | 5,25 |
| Task 2 | -0,50 | -1,05 | -0,07 | Trial number | -0,25 | -0,38 | -0,13 |
| Task 1 | -0,21 | -0,76 | 0,37 |  |  |  |  |

**Data supplements**

**Supplementary data file 1.** Latency to explore (LE), latency to feed (LF) and correct feeder choice (CE). ID 17 and 18 were not used in analysis. Times are in seconds. Task codes; s=solitary foraging, o=social task 2, observed, j=social task 1, joined.

| ID | Task | Trial | LE | LF | CE |
| --- | --- | --- | --- | --- | --- |
| 1 | s | 1 | 917 | 370 | 0 |
| 2 | s | 1 | 1 | 1084 | 0 |
| 3 | s | 1 | 37 | 5647 | 1 |
| 4 | s | 1 | 248 | 486 | 1 |
| 5 | s | 1 | 31 | 2719 | 1 |
| 6 | s | 1 | 3667 | 0 | 0 |
| 7 | s | 1 | 1 | 2 | 1 |
| 8 | s | 1 | 61 | 275 | 0 |
| 9 | s | 1 | 1 | 182 | 1 |
| 10 | s | 1 | 458 | 15 | 0 |
| 11 | s | 1 | 266 | 131 | 0 |
| 12 | s | 1 | 1 | 192 | 0 |
| 13 | s | 1 | 1 | 2749 | 1 |
| 14 | s | 1 | 1039 | 153 | 1 |
| 15 | s | 1 | 1 | 3666 | 1 |
| 16 | s | 1 | 1 | 702 | 1 |
| 19 | s | 1 | 6 | 452 | 1 |
| 20 | s | 1 | 183 | 810 | 1 |
| 21 | s | 1 | 31 | 31 | 1 |
| 22 | s | 1 | 61 | 2099 | 0 |
| 23 | s | 1 | 1 | 518 | 0 |
| 24 | s | 1 | 226 | 18 | 1 |
| 1 | s | 2 | 189 | 1 | 1 |
| 2 | s | 2 | 1 | 45 | 1 |
| 3 | s | 2 | 122 | 614 | 0 |
| 4 | s | 2 | 1 | 5 | 1 |
| 5 | s | 2 | 31 | 345 | 0 |
| 6 | s | 2 | 3667 | 0 | 0 |
| 7 | s | 2 | 1039 | 15 | 1 |
| 8 | s | 2 | 122 | 183 | 1 |
| 9 | s | 2 | 1 | 30 | 0 |
| 10 | s | 2 | 275 | 1 | 0 |
| 11 | s | 2 | 1 | 91 | 1 |
| 12 | s | 2 | 306 | 208 | 1 |
| 13 | s | 2 | 183 | 92 | 0 |
| 14 | s | 2 | 1 | 60 | 1 |
| 15 | s | 2 | 1 | 2841 | 1 |
| 16 | s | 2 | 1 | 335 | 1 |
| 19 | s | 2 | 1 | 5 | 1 |
| 20 | s | 2 | 31 | 489 | 0 |
| 21 | s | 2 | 1 | 5 | 1 |
| 22 | s | 2 | 31 | 519 | 0 |
| 23 | s | 2 | 46 | 107 | 1 |
| 24 | s | 2 | 46 | 31 | 1 |
| 1 | s | 3 | 186 | 1 | 1 |
| 2 | s | 3 | 1 | 14 | 1 |
| 3 | s | 3 | 64 | 358 | 1 |
| 4 | s | 3 | 186 | 260 | 1 |
| 5 | s | 3 | 1 | 185 | 0 |
| 6 | s | 3 | 1207 | 2154 | 1 |
| 7 | s | 3 | 177 | 52 | 1 |
| 8 | s | 3 | 1 | 11 | 1 |
| 9 | s | 3 | 1 | 885 | 1 |
| 10 | s | 3 | 114 | 69 | 1 |
| 11 | s | 3 | 244 | 95 | 1 |
| 12 | s | 3 | 1 | 30 | 1 |
| 13 | s | 3 | 1 | 198 | 1 |
| 14 | s | 3 | 76 | 92 | 1 |
| 15 | s | 3 | 672 | 244 | 1 |
| 16 | s | 3 | 1 | 14 | 1 |
| 19 | s | 3 | 3 | 12 | 1 |
| 20 | s | 3 | 9 | 6 | 1 |
| 21 | s | 3 | 1 | 2 | 1 |
| 22 | s | 3 | 46 | 61 | 1 |
| 23 | s | 3 | 244 | 214 | 1 |
| 24 | s | 3 | 76 | 1 | 1 |
| 1 | s | 5 | 92 | 61 | 1 |
| 2 | s | 5 | 1 | 253 | 1 |
| 3 | s | 5 | 1 | 152 | 1 |
| 4 | s | 5 | 15 | 34 | 1 |
| 5 | s | 5 | 122 | 12 | 1 |
| 6 | s | 5 | 73 | 156 | 0 |
| 7 | s | 5 | 15 | 52 | 0 |
| 8 | s | 5 | 1 | 195 | 1 |
| 9 | s | 5 | 1 | 17 | 1 |
| 10 | s | 5 | 168 | 21 | 1 |
| 11 | s | 5 | 153 | 159 | 1 |
| 12 | s | 5 | 1 | 2 | 1 |
| 13 | s | 5 | 1 | 305 | 1 |
| 14 | s | 5 | 1 | 30 | 1 |
| 15 | s | 5 | 1 | 855 | 1 |
| 16 | s | 5 | 1 | 14 | 1 |
| 19 | s | 5 | 6 | 3 | 1 |
| 20 | s | 5 | 18 | 12 | 1 |
| 21 | s | 5 | 15 | 46 | 1 |
| 22 | s | 5 | 1 | 2 | 1 |
| 23 | s | 5 | 1 | 2 | 1 |
| 24 | s | 5 | 31 | 18 | 1 |
| 1 | s | 6 | 24 | 61 | 0 |
| 2 | s | 6 | 6 | 43 | 0 |
| 3 | s | 6 | 1 | 5 | 0 |
| 4 | s | 6 | 61 | 83 | 0 |
| 5 | s | 6 | 92 | 92 | 1 |
| 6 | s | 6 | 31 | 174 | 1 |
| 7 | s | 6 | 1 | 2 | 1 |
| 8 | s | 6 | 1 | 2 | 1 |
| 9 | s | 6 | 1 | 14 | 1 |
| 10 | s | 6 | 138 | 290 | 1 |
| 11 | s | 6 | 122 | 257 | 1 |
| 12 | s | 6 | 1 | 366 | 1 |
| 13 | s | 6 | 1 | 152 | 1 |
| 14 | s | 6 | 1 | 11 | 1 |
| 15 | s | 6 | 1 | 1710 | 1 |
| 16 | s | 6 | 1 | 121 | 1 |
| 19 | s | 6 | 1 | 2 | 1 |
| 20 | s | 6 | 31 | 611 | 1 |
| 21 | s | 6 | 1 | 2 | 1 |
| 22 | s | 6 | 6 | 24 | 1 |
| 23 | s | 6 | 3 | 89 | 1 |
| 24 | s | 6 | 141 | 92 | 1 |
| 1 | s | 7 | 64 | 1097 | 1 |
| 2 | s | 7 | 1 | 63 | 1 |
| 3 | s | 7 | 31 | 92 | 1 |
| 4 | s | 7 | 1 | 121 | 1 |
| 5 | s | 7 | 1 | 192 | 1 |
| 6 | s | 7 | 40 | 1 | 0 |
| 7 | s | 7 | 1 | 427 | 1 |
| 8 | s | 7 | 1 | 335 | 1 |
| 9 | s | 7 | 1 | 2 | 1 |
| 10 | s | 7 | 1 | 195 | 1 |
| 11 | s | 7 | 214 | 519 | 1 |
| 12 | s | 7 | 1 | 5 | 1 |
| 13 | s | 7 | 1 | 60 | 1 |
| 14 | s | 7 | 1 | 14 | 1 |
| 15 | s | 7 | 1 | 182 | 1 |
| 16 | s | 7 | 1 | 2 | 1 |
| 19 | s | 7 | 31 | 61 | 1 |
| 20 | s | 7 | 31 | 244 | 1 |
| 21 | s | 7 | 21 | 30 | 1 |
| 22 | s | 7 | 3 | 6 | 1 |
| 23 | s | 7 | 6 | 422 | 1 |
| 24 | s | 7 | 52 | 40 | 1 |
| 1 | o | 1 | 1 | 2499 | 1 |
| 2 | o | 1 | 1 | 580 | 1 |
| 3 | o | 1 | 1 | 671 | 1 |
| 4 | o | 1 | 1 | 626 | 1 |
| 5 | o | 1 | 1 | 335 | 1 |
| 6 | o | 1 | 1 | 30 | 1 |
| 7 | o | 1 | 1 | 946 | 1 |
| 8 | o | 1 | 1 | 2499 | 1 |
| 9 | o | 1 | 1 | 732 | 1 |
| 10 | o | 1 | 1 | 183 | 1 |
| 11 | o | 1 | 1 | 733 | 1 |
| 12 | o | 1 | 1 | 1099 | 1 |
| 13 | o | 1 | 3 | 853 | 1 |
| 14 | o | 1 | 2 | 118 | 1 |
| 15 | o | 1 | 1 | 2499 | 1 |
| 16 | o | 1 | 2 | 2499 | 1 |
| 19 | o | 1 | 2 | 44 | 1 |
| 20 | o | 1 | 2 | 2498 | 1 |
| 21 | o | 1 | 1 | 45 | 1 |
| 22 | o | 1 | 1 | 66 | 1 |
| 23 | o | 1 | 2 | 555 | 1 |
| 24 | o | 1 | 2 | 328 | 1 |
| 1 | o | 2 | 1 | 803 | 1 |
| 2 | o | 2 | 1 | 182 | 1 |
| 3 | o | 2 | 1 | 427 | 1 |
| 4 | o | 2 | 1 | 75 | 1 |
| 5 | o | 2 | 1 | 305 | 1 |
| 6 | o | 2 | 1 | 188 | 1 |
| 7 | o | 2 | 1 | 1068 | 1 |
| 8 | o | 2 | 1 | 518 | 1 |
| 9 | o | 2 | 1 | 228 | 1 |
| 10 | o | 2 | 2 | 640 | 1 |
| 11 | o | 2 | 1 | 580 | 1 |
| 12 | o | 2 | 1 | 45 | 1 |
| 13 | o | 2 | 2 | 288 | 1 |
| 14 | o | 2 | 1 | 158 | 1 |
| 15 | o | 2 | 1 | 1563 | 1 |
| 16 | o | 2 | 1 | 198 | 1 |
| 19 | o | 2 | 2 | 35 | 1 |
| 20 | o | 2 | 2 | 2498 | 1 |
| 21 | o | 2 | 1 | 97 | 1 |
| 22 | o | 2 | 1 | 2499 | 1 |
| 23 | o | 2 | 1 | 436 | 1 |
| 24 | o | 2 | 2 | 618 | 1 |
| 1 | j | 1 | 2 | 2498 | 1 |
| 2 | j | 1 | 1 | 5 | 1 |
| 3 | j | 1 | 1 | 555 | 1 |
| 4 | j | 1 | 1 | 1023 | 1 |
| 5 | j | 1 | 1 | 396 | 1 |
| 6 | j | 1 | 1 | 1 | 1 |
| 7 | j | 1 | 1 | 2499 | 1 |
| 8 | j | 1 | 1 | 641 | 1 |
| 9 | j | 1 | 1 | 2499 | 1 |
| 10 | j | 1 | 1 | 2499 | 1 |
| 11 | j | 1 | 1 | 183 | 1 |
| 12 | j | 1 | 1 | 355 | 1 |
| 13 | j | 1 | 1 | 33 | 1 |
| 14 | j | 1 | 1 | 2499 | 1 |
| 15 | j | 1 | 2 | 1349 | 1 |
| 16 | j | 1 | 2 | 2499 | 1 |
| 19 | j | 1 | 1 | 137 | 1 |
| 20 | j | 1 | 1 | 2499 | 1 |
| 21 | j | 1 | 1 | 179 | 1 |
| 22 | j | 1 | 1 | 179 | 1 |
| 23 | j | 1 | 1 | 6 | 1 |
| 24 | j | 1 | 2 | 331 | 1 |
| 1 | j | 2 | 1 | 1267 | 1 |
| 2 | j | 2 | 1 | 213 | 1 |
| 3 | j | 2 | 1 | 274 | 1 |
| 4 | j | 2 | 1 | 1313 | 1 |
| 5 | j | 2 | 1 | 824 | 1 |
| 6 | j | 2 | 1 | 335 | 1 |
| 7 | j | 2 | 5 | 118 | 1 |
| 8 | j | 2 | 1 | 305 | 1 |
| 9 | j | 2 | 1 | 30 | 1 |
| 10 | j | 2 | 1 | 335 | 1 |
| 11 | j | 2 | 2 | 1190 | 1 |
| 12 | j | 2 | 1 | 152 | 1 |
| 13 | j | 2 | 2 | 2498 | 1 |
| 14 | j | 2 | 1 | 14 | 1 |
| 15 | j | 2 | 2 | 976 | 1 |
| 16 | j | 2 | 2 | 50 | 1 |
| 19 | j | 2 | 1 | 15 | 1 |
| 20 | j | 2 | 1 | 1405 | 1 |
| 21 | j | 2 | 1 | 58 | 1 |
| 22 | j | 2 | 2 | 380 | 1 |
| 23 | j | 2 | 1 | 1 | 1 |
| 24 | j | 2 | 1 | 97 | 1 |
| 1 | j | 3 | 1 | 641 | 1 |
| 2 | j | 3 | 1 | 152 | 1 |
| 3 | j | 3 | 1 | 610 | 1 |
| 4 | j | 3 | 2 | 60 | 1 |
| 5 | j | 3 | 2 | 1190 | 1 |
| 6 | j | 3 | 1 | 457 | 1 |
| 7 | j | 3 | 1 | 30 | 1 |
| 8 | j | 3 | 1 | 30 | 1 |
| 9 | j | 3 | 1 | 30 | 1 |
| 10 | j | 3 | 1 | 335 | 1 |
| 11 | j | 3 | 1 | 855 | 1 |
| 12 | j | 3 | 1 | 2499 | 1 |
| 13 | j | 3 | 1 | 134 | 1 |
| 14 | j | 3 | 1 | 21 | 1 |
| 15 | j | 3 | 2 | 380 | 1 |
| 16 | j | 3 | 2 | 136 | 1 |
| 19 | j | 3 | 1 | 103 | 1 |
| 20 | j | 3 | 2 | 304 | 1 |
| 21 | j | 3 | 1 | 57 | 1 |
| 22 | j | 3 | 2 | 227 | 1 |
| 23 | j | 3 | 1 | 2 | 1 |
| 24 | j | 3 | 2 | 115 | 1 |
